# Tecla: a telephone- and text-message based telemedical concept for patients with severe mental health disorders – study protocol for a controlled, randomized, study

**DOI:** 10.1186/s12888-015-0659-7

**Published:** 2015-11-04

**Authors:** Ulrike Stentzel, Hans-Jörgen Grabe, Lara Strobel, Peter Penndorf, Jens Langosch, Harald J. Freyberger, Wolfgang Hoffmann, Neeltje van den Berg

**Affiliations:** Institute for Community Medicine, University Medicine Greifswald, Ellernholzstraße 1-2, 17487 Greifswald, Germany; Department of Psychiatry and Psychotherapy, University Medicine Greifswald, Ellernholzstraße 1-2, 17487 Greifswald, Germany; Bethanien Hospital for Psychiatry, Psychosomatics and Psychotherapy, Gützkower Landstraße 69, 17489 Greifswald, Germany

**Keywords:** Mental health disorders, Schizophrenia, Psychotic disorders, Bipolar disorders, Telemedical care, Telemedicine, Telephone, Text messages

## Abstract

**Background:**

Severe mental disorders like psychotic disorders including schizophrenia and schizoaffective disorders have a 12-month-prevalence of 2.6, bipolar disorders of 1.5 % in Germany. The relapse risk is high; so many patients need intensive monitoring and lifelong treatment. A high medication adherence is essential for a successful treatment. But in practice, medication adherence is low and decreases over time. Telemedical care concepts might improve treatment and bridge gaps between in- and outpatient treatment.

A telemedical care concept based on regular telephone calls and short text messages was developed. The primary objective is to assess whether regular telephone calls and text messages can improve the medication adherence of patients. Secondary objectives are the reduction of rehospitalization rates, the improvement of quality of life and of the severity of symptoms.

**Methods/design:**

The Tecla study (Post stationary telemedical care of patients with severe psychiatric disorders) is a two-armed prospective randomized controlled trial. The participants in the intervention group receive in addition to usual care regular telephone calls every 2 weeks and weekly text messages on patient-individual topics during a 6 months period. Patients in the control group receive only regular care. Inclusion criteria are a physician-diagnosed bipolar disorder, schizoaffective disorder or schizophrenia and a signed informed consent. Exclusion criteria are planned inpatient treatments within the next 6 months and being non-reachable by phone. After 3 and 6 months both groups receive follow up assessments.

**Discussion:**

The primary objective of this study is the medication adherence that is measured with the Medication Adherence Report Scale, German version (MARS-D). The MARS-D is a self-report with five items. Adherent behaviour is mostly overestimated using self-reports. The strength of the MARS-D is to detect non-adherent behaviour. The original Medication Adherence Report Scale in English language (MARS-5) was developed to encourage the patient to answer truthfully to the questions that are asked in a non-threatening and non-judgmental way to minimize social desirability bias in admitting non-adherent behaviour.

**Trial registration:**

This study is registered at 2015\05\21at the German Clinical Trials Register DRKS00008548.

## Background

### Epidemiology

Nineteen new schizophrenia-cases per 100,000 people per year are diagnosed in Germany [1]. Prevalence rates of mental disorders are high all over the world, also in Europe and Germany. The lifetime prevalence of schizophrenia worldwide is estimated as 1 % of the population. The 12-month-prevalence of schizophrenia and other psychotic disorders in the German population is about 2.6 %; the 12-month-prevalence of bipolar disorders about 1.5 % [2]. In Germany 2 to 4 % of the total health care costs are caused by schizophrenic disorders [1]. Mental disorders are associated with a high disease burden. People with current mental disorders have three times as much days of limitation than people without any mental disorder [2]. Worldwide, schizophrenia is one of the ten diseases with the highest number of years of life lived with disability (YLD) [1]. This also leads to high social costs [3] because mental disorders are the most common cause for early retirement, work losses and reduced work productivity [4]. The restricted participation in social and professional life often causes a severe reduction of quality of life [1].

### Utilization and adherence

Treatment rates of mental disorders in general are low. The Study of Health in Pomerania (SHIP) [5] revealed a treatment rate for mental disorders of 20 % [6]. In the Mental Health Supplement of the German National Health Interview and Examination Survey (GHS-MHS) treatment rates for psychotic disorders (including schizophrenia) of 30.3 % of the cases and of 82.4 % for highly co-morbid cases were found. Patients with bipolar disorders received treatment in 42.2 % of the cases (62.4 % treatment in highly co-morbid cases) [7].

The diagnosis of schizophrenia implies in most cases a lifelong medical treatment. In the acute stages of schizophrenia, therapy is applied in the hospital. In this stage, drug therapy is essential to achieve a rapid and effective reduction of acute psychotic symptoms. After discharge from the hospital, adherence to medication is essential to avoid relapses. However, non-adherence is one of the major problems in patients with schizophrenia [8]. The proportion of adherent patients is 35–50 % in patients with schizophrenia and bipolar disorders [9–11]. In the outpatient setting, the adherence of patients taking atypical antipsychotics drops to 55 % after 12 months [12]. Non-adherence is associated with a higher risk of psychiatric hospitalizations [13]. Without drug therapy the relapse rate is about 70 % in the first year and about 80 % in the second year after starting drug treatment [14–16]. With drug therapy the recurrence rate is significantly lower: about 30 % in the first year and about 50 % in the second year [1].

In total 93 % of the patients with schizophrenia in Germany are treated in outpatient psychotherapeutic facilities (e.g. practices, psychiatric walk-in clinics or day-care). Especially in rural regions, waiting lists are long. A survey among practicing psychotherapists in Germany showed that adult patients have to wait on average 1.9 months for an initial interview and 4.6 months for psychotherapy [17].

### Telemedical care

Telemedical concepts have a potential to support the treatment of psychiatric patients and to provide new options in patient care. Several studies have evaluated the possibilities and limitations of telemedical care in the treatment of patients with schizophrenia. Salzer et al. [18] conducted a study with *n* = 32 schizophrenic patients consisting of brief weekly telephone-based interviews. The results showed that telephone interventions are acceptable and feasible. Because of the small study sample, these results were seen as preliminary data.

Leach and Christensen performed a systematic review about telephone-based interventions for patients with mental disorders (six in the area of depression, three of anxiety, three of eating disorders, one of substance abuse and one of schizophrenia). Although the included studies all had small simple sizes, they showed positive results [19].

Alvarez-Jimenez et al. conducted a systematic review examining the usability, acceptability, feasibility, safety or efficacy of user-led, internet- or mobile-based interventions with patients of the schizophrenia-disorders spectrum. Twelve studies were included. Patients mainly used the internet and mobile phone based interventions. The authors conclude that online, social media and mobile technologies were acceptable and feasible for patients [20].

Kasckow et al. summarized a systematic review including 18 studies with telephone-based, internet-based or video-based telehealth systems for patients with schizophrenia, that telephone, internet and video-interventions appears to be feasible [21].

Improving medication adherence using telemedical methods was discussed in several studies. In 2008 a randomized controlled trial was published that examined telephone intervention problem solving (TIPS) for patients with schizophrenia [22]. These telephone interventions were initiated weekly by telenurses and improved significantly the patient’s adherence to their psychiatric medication. Adherence was measured by pill counting and record review. The participants in the intervention showed an adherence of on average 80 %, while in control participants this rate was 60 %.

A study in Spain examined a telephone based strategy to improve adherence to antipsychotic treatment in schizophrenia. Nine hundred twenty-eight patients received a monthly telephone call by a nurse to assess therapeutic adherence and subjective attitude towards medication over a period of 4 months. Being adherent was defined as the intake of at least 60 % of the prescribed medication dose. Patients in the intervention group (*n* = 410) were significantly more adherent (OR = 3.3 95 % CI 1.6–6.6, *P* = 0.0001) than patients in the control group (*n* = 402) [23].

The study Mobile Assessment and Treatment for Schizophrenia (MATS) with 55 participating patients examined the use of mobile phone text messaging. 6 days a week individualized text messages were sent to the participants addressing the topics medication adherence, social activities and auditory hallucinations during the 12-week-intervention-period. Participants who were living independently achieved a significant improvement in medication adherence and benefited more than participants living in assisted living facilities. This is probably due to the fact, that the participants in assisted living facilities already have support and assistance in taking medications. This study was a pilot study without a control group [24].

Summarized, first studies with telemedicine concepts for patients with schizophrenia show positive results. However, evidence based on methodically sound studies with a sufficient number of participants is still limited.

The intention of Tecla study (Post stationary telemedical care of patients with severe psychiatric disorders) is to improve medical care for patients with severe mental disorders in Western-Pomerania/Germany using a telemedical intervention concept on the basis of regular, individualized telephone calls and text-messages.

## Methods/design

### The Tecla study

The outlined study Tecla is designed according to the SPIRIT Guidelines [25]. Tecla is a cooperation between the Institute for Community Medicine and the Department of Psychiatry and Psychotherapy, both University Medicine Greifswald, and the Bethanien Hospital for Psychiatry, Psychosomatics and Psychotherapy Greifswald gGmbH. An Integrated Telemedicine Centre is affiliated to the Institute for Community Medicine [26–28].

The study region Western-Pomerania is located in the very northeast of Germany in the federal state of Mecklenburg-Western Pomerania. It is a sparsely populated rural region with 460,000 inhabitants. Both in- and outpatient psychiatric and psychotherapeutic facilities and walk-in clinics are mostly located in the larger towns of the region.

### Research objectives

Primary objective of the Tecla Study is a better medication adherence after 6 months of additional telemedical treatment in the intervention group compared to a control group receiving usual care.

Secondary outcomes are a lower proportion of rehospitalized participants after 6 months, a longer time without unplanned rehospitalization, an improvement in quality of life and an improvement in the severity of the psychopathology.

Additionally, the acceptance of the telephone and text message interventions by the participants will be evaluated in the intervention group.

### Study design

**Fig. 1:**
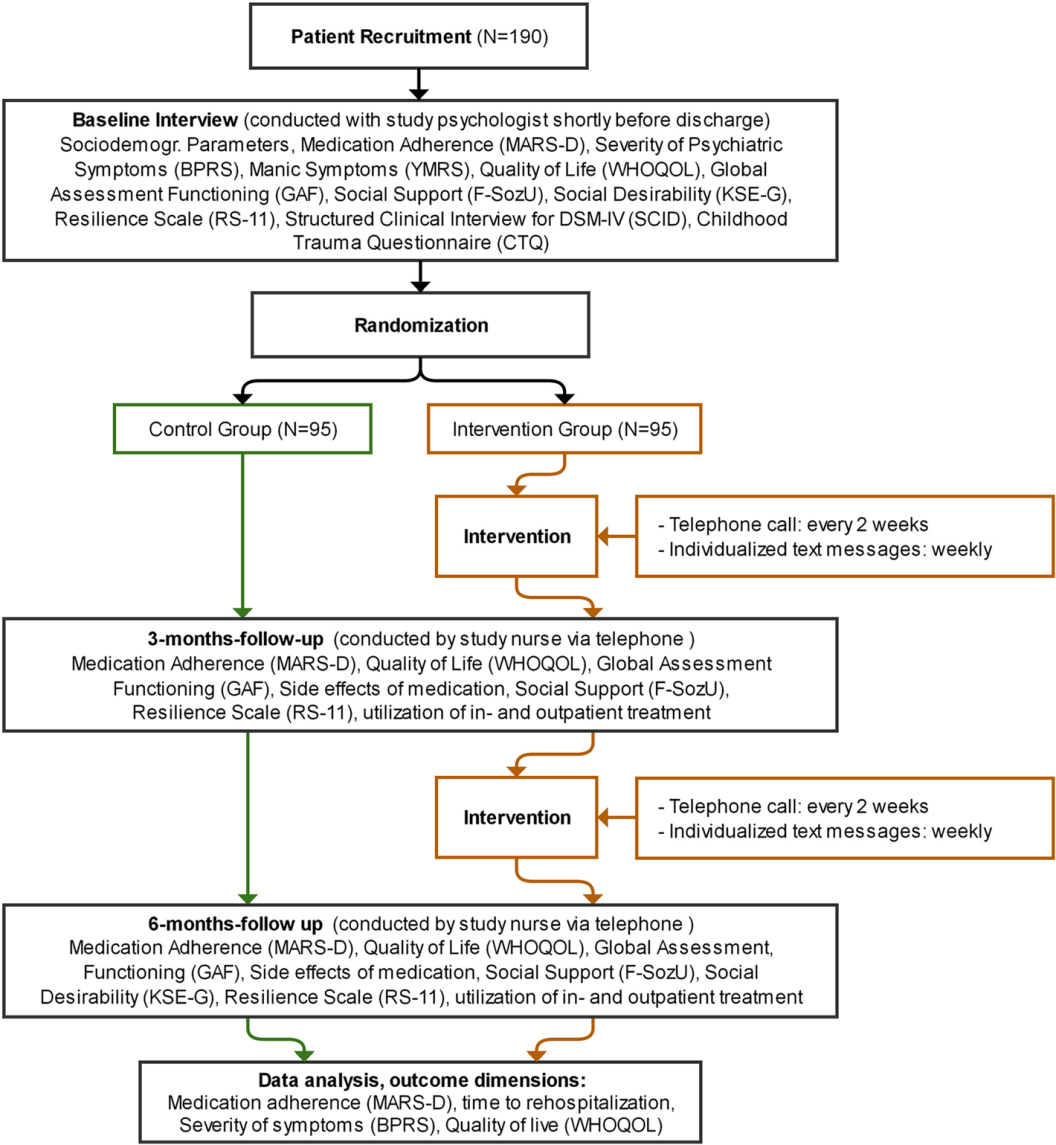
Flow chart of the study

The Tecla study is a controlled randomized prospective intervention study. Participants are recruited shortly before their discharge from the psychiatric hospital after inpatient or combined inpatient/outpatient treatment. All participants receive a comprehensive baseline interview with a study-psychologist. After the baseline interview the participants are assigned at random to the intervention or control group. Participants in the intervention group receive phone calls every 2 weeks conducted by specially trained nurses as well as weekly individualized text messages additional to usual care. The duration of the intervention period is 6 months. The participants in the control group receive usual care. After baseline, a first follow-up assessment is conducted after 3 months. The final follow-up is conducted after 6 months. Both follow ups are conducted by telephone. The structure of the study is shown in a flow chart in Fig. 1.

### Power/sample size

The sample size estimate was based on the medication adherence, measured as a score with the questionnaire MARS-D. Given an adherent behaviour in the intervention group of 80 % (corresponding to a mean score of 19) and a lower adherent behaviour in the control group of 60 % (corresponding to a mean score of 15) after 6 months, a total sample size of 128 is needed (standard deviation 8.0 for both, alpha = 0.05, power = 0.80). Assuming a lost to follow up about 50 %, we will include 190 patients to the study.

### Recruitment and participants

Patients are recruited from three psychiatric hospitals in the region shortly before their discharge. The inclusion criteria are met if the patient is at least 18 years old, has a medical diagnosis in the field of bipolar disorders (ICD-10 F31), schizoaffective disorders (ICD-10 F25) or any form of schizophrenia (ICD-10 F20). Patients with already scheduled inpatient treatments within the next 6 months are excluded. In addition, patients can’t participate, if they are not reachable by phone. The study psychologist visits the participating hospital departments to inform the patients on site and invite them to participate in the study. All patients have to give written informed consent. Therein the patients declare consent to

- be contacted by the study nurse over a period of 6 months by call and by text messages
- to the scientific evaluation of the collected data in a pseudonymised form
- to be possibly contacted again after the participation in the project to ascertain how further treatment is progressing
- the use of further data (like data from hospital information systems about other in- or outpatient treatments or data of the statutory health insurance) in the evaluation.

Patients can withdraw their consent or parts of it anytime and without giving reasons. The withdrawal does not create disadvantages.

The recruitment phase of the project started February 2015.

### Baseline and follow-up interviews

Shortly before their discharge from the hospital, all participants receive a baseline interview conducted on site by the study psychologist. The baseline interview consists of the assessment of personal data (name, address, telephone number, date of birth), diagnoses, medication, by the patients’ desired topics for the telephone calls and several standardized questionnaires covering the outcomes of the study. The diagnoses are taken from the medical record. In addition the Structured Clinical Interview for DSM-IV (SCID I) is performed, but only the specific sections about affective disorders and the sections about the psychotic disorders [29]. Topics for telephone calls selected from a list by the patient are medication, housing/living, work, finances, spare time, friends, family, partnership, everyday life, diseases and others.

The medication adherence is measured using the Medication Adherence Report Scale, German version (MARS-D) [30]. The MARS-D consists of five items, detecting non-adherent behaviour by self-report. It is known that patients tend to overestimate their adherence [31–33] or to conceal non-adherent behaviour [30]. The MARS-5 (original Medication Adherence Report Scale in English language) was developed to take the frequency of non-adherent behaviour into account and to encourage the patient to answer truthfully to the questions that are asked in a non-threatening and non-judgmental way to minimize social desirability bias [34, 35].

The mental health status is measured by the Brief Psychiatric Rating Scale (BPRS). This instrument consists of 18 items to measure the severity of psychiatric symptoms [36, 37]. The Young Mania Rating Scale (YMRS) in German Language will be used for the assessment and quantification of manic symptoms [38].

Quality of life will be measured on the basis of the short version of the subjective instrument World Health Organization Quality of Life (WHOQOL-BREF), which is designed for generic use [39, 40].

The psychological, social and occupational function levels will be assessed by the Global Assessment of Functioning (GAF) on a scale from 1 to 100, intended as a hypothetical continuum of mental health (score 91–100) to illness (score 1–10) [41].

In the baseline interview the following issues will also be assessed in a standardized computer assisted personal interview:

- Socio-demographic information
- Side effects of medication according to a list of common side effects of psychotropic drugs
- Social support (F-SozU, short form with 14 items) [42]
- Short scale social desirability-gamma (KSE-G) [43]
- Resilience scale (RS-11, short form with 11 items) [44]
- Use of alcohol, drugs and smoking status [45, 46]
- Childhood Trauma Questionnaire (CTQ) [47]

The 3- and 6-months-follow-ups will be conducted by telephone by the nurses of the telemedicine centre as a computer assisted personal interview. In both follow-ups, the MARS-D, WHOQOL-BREF, the side effects of medication, social support, social desirability, the resilience scale and furthermore, changes in medication and the utilization of in- and outpatient treatment will be assessed. The utilization of other treatments will be assessed by asking whether the participants have visited a general practitioner or other specialized physicians including psychotherapist/psychiatrists within the last three respectively 6 months and if so, how often. Also the number and duration of hospital stays are questioned. The GAF will be assessed only in the 6-months-follow-up.

In the 6-months-follow-up the participants in the intervention group will also be asked how they evaluate the intervention (Table 1).

**Table 1:** Interview questions and answers to assess acceptance and satisfaction of the participants

Question:	How would you assess the telephone and text messages contacts during the last 6 months?
Answer:	Very helpful – little helpful – not helpful – other (free text) – don’t know – no answer
Question:	Could you imagine continuing the telephone contacts in this form?
Answer:	Yes – No – don’t know – no answer
Question:	Do you think this kind of care can partly replace personal contacts with physicians or psychologists?
Answer:	Yes – No – don’t know – no answer
Question:	Is there something you would change or improve?
Answer:	Yes – No – don’t know – no answer and additional free text

### Intervention

The intervention consists of telephone calls every 2 weeks and standardized weekly text messages, conducted by specially trained nurses in a telemedicine centre. Participants can send answers to the text messages or use this medium to contact the nurses. The intervals can be reduced if necessary. Additionally, the participants can contact the nurses within office hours. If appropriate, individualized text messages are being sent.

Each telephone call starts with a question about the health condition of the participants: How do you do feel today? What went well today or yesterday? Are there any problems? Afterwards the topics that were selected in the baseline interview are being discussed. These topics can be changed, if participants want to talk about other topics. After discussing the chosen topic, the participants should assess how well they performed on that topic and choose one of the following descriptions: “very well”, “well”, “satisfactory”, “deficient”, “inadequate”, “don’t know” and “no answer”.

This open conversation is followed by a standardized assessment of the following aspects:

- Changes in medication
- Side effects of the medication. The side-effects are being assessed with the descriptions “no side effects”, “little”, “moderately”, “strongly” and “very strong” for each of the following: movement disorders, muscle stiffness, involuntary shiver, motionlessness, muscle spasm, agonizing restlessness/problems to sit still (can’t be suppressed at will), lack of sexual desire/loss of libido, increase in weight, increased appetite, heavy feeling of illness/chills/fever and milk flow.
- Medication intake. Assessment of adherence (e.g. forgetting to take drugs, arbitrary changes in dose or skipping doses).
- Suicidal tendencies. The assessment of suicidal tendencies consists of asking for suicidal thoughts, concrete intentions and plans: are these thoughts imposing or conscious, talked to someone about, reduction of interpersonal contacts and interests and make contact to relatives or treating physician.

The intervention call ends with concluding remarks:

- What are you taking with you from the call today?
- Would you like to discuss another topic next time?
- Would you like to ask me something or do you have another wish yet?

At the end, an appointment for the next call is made.

After each call, based on the information solicited, the nurses rate the health conditions of the participant on the basis of an adaption of the BPRS on a range from one (minimal manifestation) to ten (maximal manifestation) for each of the items: receptiveness/cooperation, concentration, ability of vibration, Strain/nervousness, hostility, distrust/paranoid thought content, uncooperative behaviour and unusual, bizarre thoughts of the patient.

The weekly text messages take up global themes such as “I wish a good start in the week.” Or individual messages such as “You wanted to go to a choir practice. Did you go and how was it?” The participants can answer and the nurses will response if necessary or appropriate.

### Documentation, data storage, data security and data protection

The documentation of all data (baseline interview, documentation of the telephone calls and follow-up interviews) is conducted on the basis of eCRFs in an IT-supported documentation system [48]. The data is saved in a central project database. The storage is done according to the current standards for data security and data privacy [49]. These standards are documented in the institutional data protection concept of the Institute for Community Medicine and the telemedicine centre. Only the study psychologist and the nurses have access to personal data during the intervention phase. After completion the 6-month-follow-up, the data will be pseudonymised and identifying data will be separated from the project data and kept in the independent trustee’s office of the University Medicine Greifswald.

### Ethics approval

The institutional ethics committee of the University Medicine Greifswald evaluated the study, design and instruments and testified the compliance with ethical requirements at 2015\01\27 (registration number BB 122/14).

### Trial registration

The Tecla study is registered at 2015\05\21 at the German Clinical Trials Register (DRKS00008548).

### Data analysis

Data analyses will be conducted in a strictly pseudonymised way. First, a descriptive analysis will be conducted. All outcomes will be analysed as a comparison between the groups at 3 and 6 months. Acceptance will be evaluated only in the intervention group.

## Discussion

A previous study for patients with anxiety, depressive symptoms and somatization showed positive effects on the symptom scores for anxiety and depression [50]. Based on these results, the outlined study was initiated to improve the medical care for patients with severe mental disorders. In most cases a lifelong medical treatment is necessary, therefore, medication adherence is very important, but low in many cases [14-16]. Regular telephone calls and text messages might improve adherent behaviour. Medication adherence is measured with the self-report “Medication Adherence Report Scale”, German version (MARS-D) [30]. Self-reports inhere the risk of biased estimations. The original five item “Medication Adherence Report Scale” (MARS-5) was developed to minimize social desirability bias [34, 35]. Due to the risk of selective drop out, a challenging part of this study will be the performance of the follow-up examinations after 3 and 6 months, in particular with patients of the control group. Changing opinions, forgetting appointments for calls or rehospitalizations are probable reasons for lost to follow-up, especially in this patient group.

## Abbreviations

BPRS: Brief Psychiatric Rating Scale
eCRF: electronic care report forms
F-SozU: Social support questionnaire
GAF: Global assessment functioning
GHS-MHS: Mental Health Supplement of the German National Health Interview and Examination Survey
KSE-G: Short scale social desirability-gamma questionnaire
MARS-5: Original Medication Adherence Report Scale in English language
MARS-D: Medication Adherence Report Scale, German version
MATS: Mobile Assessment and Treatment for Schizophrenia
RS-11: Resilience scale questionnaire
SHIP: Study of Health in Pomerania
TIPS: Telephone intervention problem solving
WHO: World Health Organization
WHOQOL: World Health Organization Quality of Life
YLD: Years of life lived with disability
YMRS: Young Mania Rating Scale
